# Intracranial hemangioblastoma – A SEER-based analysis 2004–2013

**DOI:** 10.18632/oncotarget.25534

**Published:** 2018-06-15

**Authors:** Ha Son Nguyen, Ninh B. Doan, Michael Gelsomino, Saman Shabani, Ahmed J. Awad, Mayank Kaushal, Martin M. Mortazavi

**Affiliations:** ^1^ Department of Neurosurgery, Medical College of Wisconsin, Milwaukee, Wisconsin, USA; ^2^ National Skull Base Center, Thousand Oaks, California, USA; ^3^ California Institute of Neuroscience, Thousand Oaks, California, USA; ^4^ Faculty of Medicine and Health Sciences, An-Najah National University, Nablus, Palestine; ^5^ Department of Neurosurgery, University of South Alabama, Mobile, Alabama, USA

**Keywords:** intracranial hemangioblastoma, SEER, surgery, radiation

## Abstract

**Introduction:**

Intracranial hemangioblastoma (HB) is a rare pathology. Limited data exist regarding its epidemiology.

**Methods:**

With the SEER-18 registry database, information from all patients diagnosed with intracranial HB from 2004 to 2013 were extracted, including age, gender, race, marital status, presence of surgery, extent of surgery, receipt of radiation, tumor size, tumor location, and follow-up data. Age-adjusted incidence rates and overall survival (OS). Cox proportional hazards model was employed for both univariate and multivariate analyses.

**Results:**

A total of 1307 cases were identified. The overall incidence of intracranial hemangioblastoma is 0.153 per 100,000 person-years [95% confidence interval (CI)=0.145–0.162]. Through univariate analysis, age < 40 [hazard ratio (HR)=0.277, p<0.001], no radiation [HR=0.56, p=0.047], and presence of surgery [HR=0.576, p=0.012] are significant positive prognostic factors. Caucasian race [HR=1.42, p=0.071] and female gender [HR=0.744, p=0.087] exhibit noticeable trends towards positive prognosis. Through multivariate analysis, younger age [HR=1.053, p < 0.01], race [HR=1.916, p<0.01], and presence of surgery [HR=0.463, p<0.01 were significant independent prognostic factors.

**Conclusion:**

Clinical factors such as younger age, Caucasian race, and presence of surgery are significant independent factors for overall survival in patients with HBs. Though analysis regarding extent of surgery did not produce a meaningful relationship, this may be related to surgical bias / expertise. Moreover, no validation for radiation therapy was identified, but this may be related to short follow up intervals and the variable growth patterns of HBs.

## INTRODUCTION

Hemangioblastomas (HBs) are benign neoplasms (WHO grade I) that constitute roughly 2% of intracranial neoplasms and 2-10% of primary spinal cord neoplasms [1]. The pathology frequently arises below the tentorium, predominantly in the cerebellar hemispheres (up to 76%), near the brain stem, or along the spinal cord [2, 3]. These lesions can appear either sporadically (57–75%) or due to an association with von Hippel-Lindau (VHL) disease (20–43%) [3]. HBs stem from “stromal” cells of unknown origin, and comprise of vascular cell types, including endothelial and pericytes [4, 5].

Presenting symptoms are commonly related to mass effect due to tumor growth, cyst formation, and peri-tumoral edema. Surgical resection has been the mainstay treatment. Radiation therapy is also an option for less accessible lesions, for multiple concurrent lesions, and for tumor control after subtotal resection. Data regarding incidence and survival have been presented in small series. The Surveillance, Epidemiology, and End Results (SEER) Program, supported by the National Cancer Institute, amasses cancer statistics that cover nearly 28% of the United States population. Accordingly, we scrutinized the database to evaluate the epidemiology of HBs.

## RESULTS

### Epidemiology

The overall incidence of intracranial HBs within the SEER database is 0.153 per 100,000 person-years [95% confidence interval (CI) = 0.145–0.162]. The incidences for females and males are 0.132 (CI = 0.122-0.144) and 0.177 (CI = 0.164-0.190) respectively. There is a decreased incidence among female compared to males [Incidence rate ratio (IRR) = 0.7478, CI = 0.6689–0.8356, p < 0.001]. Compared to Caucasian-Americans, African-Americans (IRR = 0.8284, CI = 0.6810–1.0003), American Indians/Alaska Natives (IRR = 0.7312, CI = 0.4018–1.2505), and Asian or Pacific Islanders (IRR = 0.8418, CI = 0.6880–1.0223) all have decreased incidences, but each with p value > 0.05.

**Figure 1:**
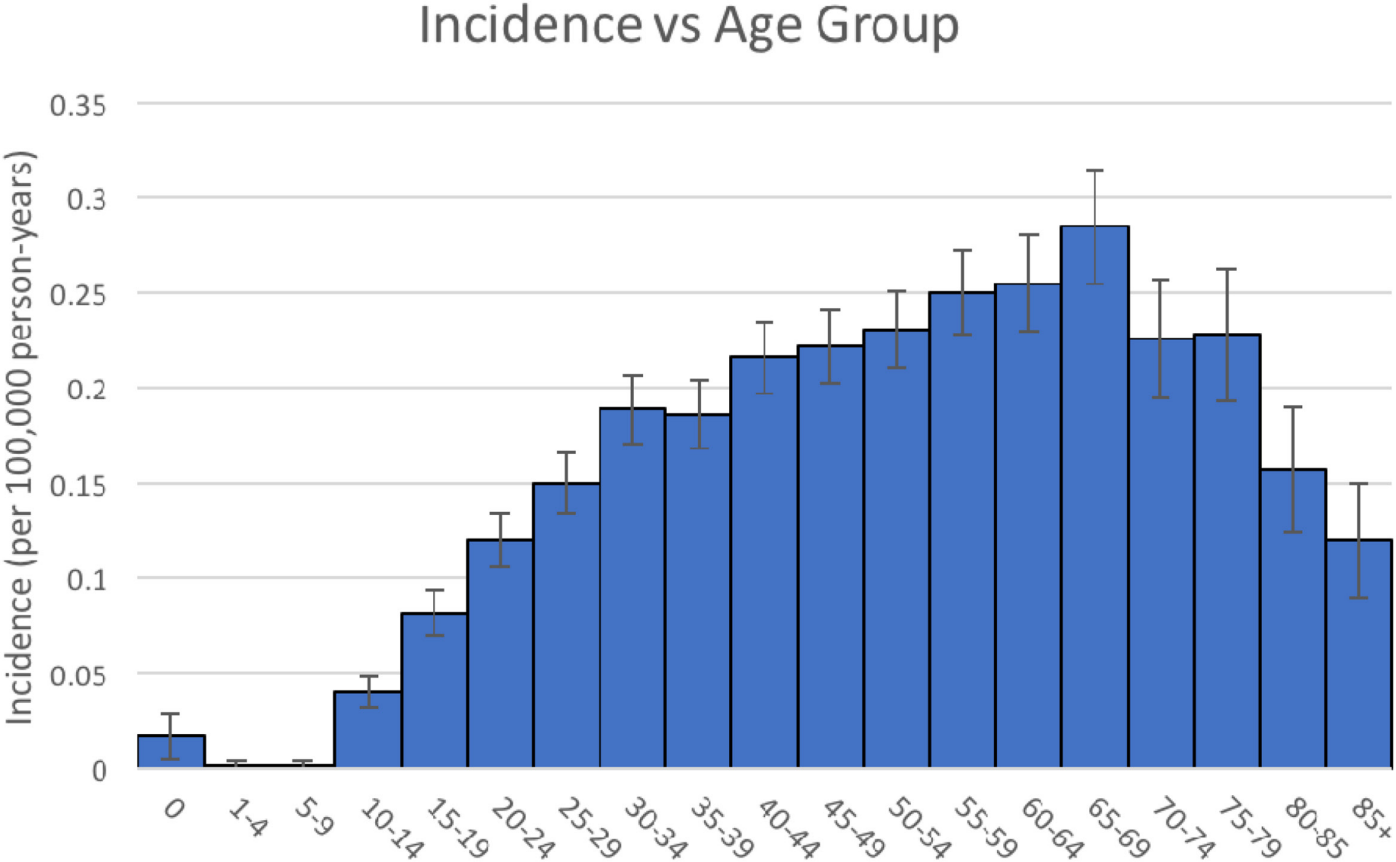
Intracranial hemangioblastoma - Incidence vs Age Group

As shown in Figure 1, the incidence of intracranial HBs remains low in the younger age groups. The incidence increases with age group 10-14 and peaks at age group 65-69 (ranging from 0.040 to 0.285 per 100,000 person-years). Values between age groups 15-19 to 85+ years are significantly different than the rate for age group 0 years (p < 0.05).

### Univariate analysis

Like prior analyses [6, 7], only cases with actively followed / known age / within research database were considered; death certificate only / autopsy only / alive with no survival time were excluded. A total of 1307 cases were identified in the SEER-18 database. Table 1 summarizes patient characteristics.

**Table 1:** Univariate analysis

		n	%	5 year OS	Univariate HR	Lower 95%	Upper 95%	p value
Age	1307							
<40		468	35.81	95.11	0.277	0.175	0.441	<0.001
40+		839	64.19	81.64				
Gender	1307							
Male		573	43.84	85.40	0.744	0.529	1.044	0.087
Female		734	56.16	88.09				
Marital status	1240							
Married		716	57.74	87.32	0.763	0.546	1.065	0.112
Not Married		524	42.26	85.04				
Race	1289							
Caucasian		1031	79.98	87.63	1.42	0.97	2.079	0.071
Non-caucasian		258	20.02	81.84				
Size	937							
<=3 cm		413	44.08	84.03	1.213	0.823	1.79	0.329
3+ cm		524	55.92	88.03				
Location	1130							
Supra-tentorial		108	9.56	84.40	0.706	0.417	1.194	0.194
Infra-tentorial		1022	90.44	86.96				
Radiation	1297							
No		1231	94.91	87.54	0.56	0.317	0.992	0.047
Yes		66	5.09	73.54				
Surgery?	1305							
No		143	10.96	79.00	0.576	0.375	0.887	0.012
Yes		1162	89.04	87.50				
GTR?	1144							
No		508	44.41	85.70	1.193	0.829	1.718	0.343
Yes		636	55.59	88.97				

The median follow-up was 47 months. The median OS was not attained. The 3-, 5-, and 9-year OS were 89.69% (CI = 88.76−90.62%), 86.56% (CI = 85.42−87.70%), and 80.18% (CI = 77.58−82.78%) respectively.

Mortality occurred in 142 patients. Of the documented causes of mortality, death was attributable to HBs for 60 patients [Brain and Other Central Nervous System (13); *in situ*, benign or unknown behavior neoplasm (42); miscellaneous malignant cancer (5)]. Other causes of death include Alzheimer's (3), aortic aneurysm and dissection (1), breast (1), cerebrovascular diseases (6), chronic obstructive pulmonary disease and allied conditions (1), congenital anomalies (5), diabetes mellitus (1), esophagus (1), diseases of the heart (20), hypertension without heart disease (1), intrahepatic bile duct (1), kidney and renal pelvis (3), lung and bronchus (6), melanoma of the skin (1), myeloma (1), ovary (1), pancreas (1), pneumonia and influenza (2), prostate (1), septicemia (2), urinary bladder (1), other infectious (2), other causes of death (11), not available (8), and ill-defined (1).

Age < 40 [hazard ratio (HR) = 0.277, p < 0.001], no radiation [HR = 0.56, p = 0.047], and presence of surgery [HR = 0.576, p = 0.012] are significant positive prognostic factors. Race [HR = 1.42, p = 0.071] and gender [HR = 0.744, p = 0.087] exhibit noticeable trends towards positive prognosis. Interestingly, extent of surgery (to GTR) is not a significant prognostic factor. Neither are marital status, size of tumor, or location of tumor. See Table 1.

### Multivariate analysis

From univariate analysis, variables with p < 0.1 (age, gender, race, presence of surgery, and receipt of radiation) were incorporated in a multivariate analysis. For this analysis, age was reverted to a continuous variable. The regression revealed that younger age [HR = 1.053, p < 0.01], race [HR = 1.916, p < 0.01], and presence of surgery [HR = 0.463, p < 0.01 were significant independent positive prognostic factors. See Table 2.

**Table 2:** Multivariate analysis

	HR	Lower 95.0%	Upper 95%	P value
Age	1.05	1.04	1.06	<0.01
Gender	0.72	0.51	1.02	0.07
Race	1.92	1.30	2.83	<0.01
Surgery (Y/N?)	0.46	0.30	0.72	<0.01

## DISCUSSION

To our knowledge, this study represents the largest cohort of patients diagnosed with intracranial HBs. The study augments the current literature regarding overall incidence, and incidence relative to age groups, gender, and race. Figure 1 depicts that pediatric cases remain scarce compared to adult cases [8]; average age in the SEER cohort was 47.09 years, comparable to prior literature where the average age at diagnosis occurs earlier in VHL disease (30–40 years) compared with sporadic cases (40–50 years) [8]. The relative male predominance in the SEER cohort was also consistent with prior observations as well. Moreover, the cohort exhibited no obvious significant difference in incidence rates among race.

Gender, marital status, tumor size, and tumor location did not demonstrate any significant relationship with overall survival. Likewise, a recent meta-analysis by Pan et al [1] discovered similar patterns with these factors. From our univariate analysis and multivariate analysis, younger age was a significant independent prognostic factor for overall survival. Fukuda et al [9] noted a similar finding when the group assessed the outcomes of 36 patients who underwent surgical resection of sporadic HBs. Various studies assessing radiation treatment also noted this pattern [10]. Race appears to be a significant prognostic factor based on the multivariate analysis. Caucasian patients exhibited a longer overall survival then non-Caucasian patients. Overall, this finding is congruent with other population-based studies that have utilized the SEER registry. Suggested reasoning includes differences with biology (molecular or genetic factors) and/or with socioeconomics (access to routine health care) [11–15].

Our analysis revealed that the presence of surgery also increased overall survival. Interestingly, sub-analysis regarding extent of surgery (to GTR) did not demonstrate statistical significance. This counters the pervasive, ongoing, clinical notion that extent of resection improves clinical outcome [10], especially given the high reported rates of recurrence or progression (up to 25-40%) [16]. The result may be a combination of a few issues. Within the SEER cohort, the percentage of patients who underwent surgery was high (89%), but the proportion with GTR was low (~56%) compared to prior case series (up to ~89%) [3]. Since the SEER registry includes non-academic facilities, but prior literature arises predominately from academic institutions, the lower GTR may reflect a bias for comfort level / surgical expertise. Moreover, the decision for surgery remains controversial. Some surgeons may decide to operate based on radiographic progression, while others wait for development of symptoms. For instance, VHL-associated pathology may be uncovered early, but elicits no symptoms. Ammeran et al [17] followed 19 VHL patients with 143 tumors, and noted that although all patients exhibited radiographic progression, only 41% displayed symptoms. Besides, the growth rate may be dependent on location of the tumor as well. While reviewing supratentorial HBs, Peyre et al [18] noticed that growth rates, which may be slow or rapid, can remain relatively steady over time. On the other hand, cerebellar and spinal HBs can display a stuttering growth pattern, with intervals of growth (mean 13 ± 15 months) followed by intervals of latency (mean 25 ± 19 months) [1, 18].

Only approximately 5% underwent radiation in this SEER cohort. On univariate analysis, receipt of radiation was associated with decreased overall survival. However, accounting for other qualifying factors in the multivariate analysis, receipt of radiation was not a significant independent factor. Due to the variable growth patterns of HBs, Tomasello et al [19] emphasized the difficulty associated with short-term follow-ups, which was a common feature in most studies; the lack of tumor growth could be associated with a latent growth phase or the effect of radiation. Recent studies have shown that radiation is a viable treatment option. Kano et al [10] published an international, multicenter, retrospective study of 189 patients with 517 tumors regarding stereotactic radiosurgery, purporting local control rates of 92% at 3 years, 89% at 5 years, and 79% at 10 years, with overall survival rates of 94% at 3 years, 90% at 5 years, and 74% at 10 years. A recent meta-analysis, which incorporated this study and several others, also reported progression free survival at 88.4% [1]. Adverse consequences of stereotactic radiosurgery (reported median 3.1%) [1] include hydrocephalus, peri-tumoral swelling, and radiation necrosis.

This study has limitations that are built-in to SEER analysis. As Bates et al [20] outlined, detailed information regarding radiation (dosage, fields, or fractionation) are not collected. Early studies have suggested that local tumor control rates were positively correlated with the radiation dose [1]. Similarly, pertinent surgical details are not presented. Utilization of preoperative embolization [21, 22], tumor consistency [1, 9, 16, 23], preoperative status [10], and postoperative morbidity [3, 24, 25] have been associated with poor outcomes in HBs. Moreover, data regarding disease progression are lacking. Median follow-up was only 47 months in the database; since the pathology is relatively benign and median survival was not reached, extended follow-up could elicit noteworthy correlations [20]. This can be particularly relevant with HBs. The database did not differentiate between sporadic HBs and VHL associated HBs. The latter has a different underlying pathophysiology and requires routine surveillance. Nevertheless, prior studies regarding the influence of VHL status on tumor progression and overall outcomes has been conflicting [1, 3, 10].

## MATERIALS AND METHODS

### Patient cohort

This analysis was done in a similar manner to prior literature [6, 7, 20]. Through the SEER-18 registry (including Hurricane Katrina impacted Louisiana), we screened for patients with HBs registered from 2004 to 2013 [26]. All cases assigned the ICD-O-3 histologies codes 9161/1 (hemangioblastoma) and 9161/3 (hemangioblastoma, malignant) were included in this analysis. Patients with prior malignancies were included. Those involving spinal cord (C72.0), cauda equina (C72.1), or spinal meninges (C70.1) were excluded. Age, gender, race, presence of surgery, extent of primary surgery, receipt of radiation, tumor size, and follow-up data were obtained. Primary site was determined via the ICD-O-3 site code.

### Statistics

With the SEER^*^Stat software, the following was performed similar to previously described [6, 7, 20]: 1) age-adjusted incidence rates were computed as the number of HB cases per 100,000 person-years, 2) The effect of age, gender, and race on incidence was also analyzed, where incidence rate ratios (IRRs) were computed with a significance threshold p = 0.05, and 3) Overall survival (OS) were computed using the Kaplan–Meier method. Causes of death coded as “brain and other nervous system,” “*in situ*, benign, or unknown behavior neoplasm,” and “miscellaneous malignant cancer” were all attributed to HBs, as previously utilized for other SEER analyses [7, 20]. Categorical data are depicted with frequency counts and percentages.

Age was dichotomized using a threshold of 40 years of age for univariate analysis, and treated as a continuous variable for multivariate analysis. Gender was dichotomized to males and females. Marital status was dichotomized to single (coded as “single, never married”, “separated”, “divorced”, “widowed”, or “unmarried or domestic partner”) and married (coded as “married, including common law). Race was dichotomized to Caucasian (coded as “white”) or Non-Caucasian (coded as “black”, “American Indian / Alaska Native”, or “Asian or Pacific Islander.” Tumor size was dichotomized using a threshold of 3 cm. A patient with an unknown value for the specific variable was excluded from the analysis of that specific variable.

Location of tumor was dichotomized to “supratentorial” – those coded as C71.0 Cerebrum, C70.0 Cerebral meninges, C71.1 Frontal lobe, C71.2 Temporal lobe, C71.3 Parietal lobe, C71.4 Occipital lobe, C72.3 Optic nerve, OR “infratentorial” – those coded as C71.6 Cerebellum NOS, C71.7 Brain stem, C72.4 Acoustic nerve. Unknown or unclear locations – those coded as C71.9 Brain, NOS, C72.9 Nervous system NOS, C71.5 Ventricle NOS, C71.8 Overlapping lesion of brain, C72.8 Overlapping lesion of brain and CNS, C72.5 Cranial nerve NOS – were not included in the location analysis.

Presence of surgery was defined as follows: “No surgery” – those coded as “no surgery (00)” OR “Surgery” – those coded as local tumor destruction NOS (10), biopsy (20), surgery NOS (90), partial resection NOS (40), and subtotal resection (21), gross total resection (55), or radical, total, gross total resection (30). Of those who underwent surgery, the extent of primary surgery was defined as follows, similar to previously described [7, 27]: “No GTR” – those coded as “local tumor destruction NOS (10), biopsy (20), partial resection NOS (40), and subtotal resection (21)” OR “GTR” – those coded as gross total resection (55) or radical, total, gross total resection (30). “Surgery status unknown” – those coded as surgery, unknown (99) – and surgery NOS (90) was not included in the extent of resection analysis.

Receipt of radiation was defined as follows: “No radiation” – those coded as none (0) and patient or patient's guardian refused radiation therapy (7) OR “Radiation” – those coded as beam radiation (1), radioactive implants (2), radioisotopes (3), combination of 1 with 2 or 3 (4), and radiation NOS (5); unknown status of radiation was not included in the relevant analysis.

IBM SPSS 22 was utilized for statistical analysis. For univariate analysis, relationships between various demographic / treatment variables and OS were determined using the Cox proportional hazards model. Those that exhibited p < 0.1 were incorporated into a backwards-conditional multivariate analysis that also employed the Cox proportional hazards model. All p values reported represent two-sided statistical tests. A p < 0.05 were considered statistically significant.

## CONCLUSIONS

Clinical factors such as younger age, race, and presence of surgery are significant independent factors for overall survival in patients with HBs. Though analysis regarding extent of surgery did not produce a meaningful relationship, this may be related to surgical bias / expertise. Moreover, no validation for radiation therapy was identified, but this may be related to short follow up intervals and the variable growth patterns of HBs.

## Abbreviations

CI: Confidence Interval
GTR: Gross total resection
HB: Hemangioblastoma
HR: Hazards ratio
IRR: Incidence rate ratio
OS: Overall survival
SEER: Surveillance Epidemiology
VHL: von Hippel-Lindau
